# Acute Strenuous Exercise Induces an Imbalance on Histone H4 Acetylation/Histone Deacetylase 2 and Increases the Proinflammatory Profile of PBMC of Obese Individuals

**DOI:** 10.1155/2017/1530230

**Published:** 2017-10-19

**Authors:** Gilson P. Dorneles, Maria Carolina R. Boeira, Lucas L. Schipper, Ivy R. V. Silva, Viviane R. Elsner, Pedro Dal Lago, Alessandra Peres, Pedro R. T. Romão

**Affiliations:** ^1^Graduate Program in Health Sciences, Laboratory of Cellular and Molecular Immunology, Federal University of Health Sciences of Porto Alegre, 90050-170 Porto Alegre, RS, Brazil; ^2^Research Center, Graduate Program in Bioscience and Rehabilitation, Methodist University Center IPA, 90420-060 Porto Alegre, RS, Brazil; ^3^Graduate Program in Rehabilitation Sciences, Federal University of Health Sciences of Porto Alegre, 90050-170 Porto Alegre, RS, Brazil

## Abstract

This study evaluated the response of global histone H4 acetylation (H4ac), histone deacetylase 2 (HDAC2) activity, as well as the production of proinflammatory cytokines and monocyte phenotypes of lean and obese males after exercise. Ten lean and ten obese sedentary men were submitted to one session of strenuous exercise, and peripheral blood mononuclear cells (PBMC) were stimulated *in vitro* with lipopolysaccharide **(**LPS**)**. Global H4ac levels, HDAC2 activity in PBMC, and IL-6, IL-8, and TNF-*α* production were analyzed. Monocyte phenotype was determined in accordance with the expression of CD14 and CD16. At rest, obese individuals presented higher frequency of proinflammatory CD14^+^CD16^+^ monocytes. LPS induced a significant augment in global H4ac and in the production of IL-6, IL-8, and TNF-*α* mainly in obese individuals. After exercise, the increased production of IL-8 and TNF-*α* and peripheral frequency of CD14^+^CD16^+^ were observed in both groups. In addition, exercise also induced a significant hyperacetylation of histone H4 and decreased HDAC2 activity in both nonstimulated and LPS-stimulated PBMC of obese individuals. Our data indicate that the obesity impacts on H4ac levels and that strenuous exercise leads to an enhanced chronic low-grade inflammation profile in obesity via an imbalance on H4ac/HDAC2.

## 1. Introduction

Obesity is considered an epidemic disease in many developed and developing countries, where it has become a major public health problem [1]. In obese people, the chronic low-grade inflammation is linked to cardiometabolic diseases, such as diabetes and atherosclerosis, and risk of premature death [2, 3]. Early studies have described that this proinflammatory condition is associated with high expression of nuclear factor kappa-B (NF-*κ*B) in peripheral blood mononuclear cells (PBMCs) [4, 5]. In fact, obesity is associated with a proinflammatory profile of immune cells, specially monocytes/macrophages and type 1 T helper (Th1) cells and an elevation in the adipose tissue expression and plasma concentrations of several inflammatory mediators including tumor necrosis factor-alpha (TNF-*α*) and interleukin-6 (IL-6) [3, 4, 6].

Emerging evidences indicate that epigenetic events play a pivotal role in the interplay between genes and environmental factors that generate the inflammatory pathway in obesity [7, 8]. Consequently, the epigenetic regulation, in particular, posttranslation histone modifications have a pivotal role in inflammation and immune response [7]. Histone acetylation occurs by histone acetyltransferases (HATs). Conversely, histone deacetylases (HDACs) remove the acetyl groups from the amino-terminal tail of acetylated histones, counteract the effects of HATs, and return histone to its basal state [9]. Moreover, the acetylation of histone H4 (H4ac) is especially important for chromatin structure, gene expression, cell polarization, and cytokine production [10, 11]. In this regarding, it was demonstrated that the lipopolysaccharide- (LPS-) stimulated monocytes/macrophages M1 polarization and the expression of NF-*κ*B are dependent of global H4ac status [12–14]. In a genome-wide study, Zhang and coworkers [12] described that in M1 macrophages, the H4ac expression was significantly increased after stimulation with interferon-*γ* (IFN-*γ*), but little was modified by the IL-4 treatment. In addition, it was shown that the activation of NF-*κ*B and the release of TNF-*α* and IL-8 by cultured cells was widely dependent on H4ac, confirming that the acetylation levels and enhanced transcriptional activity are able to modulate the cytokine production during endotoxin stimulation [13, 15]. The H4ac status is controlled by a variety of HDAC isoform enzymes that reduces the transcription of inflammatory genes in monocytes [16]. Among a series of HDAC subtypes, it was shown that the transactivation of NF-*κ*B and TNF-*α* expression was downregulated through the interaction of p65 subunit of NF-*κ*B with HDAC1 and HDAC2 [17]. Moreover, Ito and coworkers [18] have described that the HDAC2 reduces the acetylation of histone H4 at lysines 8 and 12 and the production of IL-1*β*. However, there is no data about the influence of obesity on H4ac status and HDAC2 activity.

Physical exercise is considered a subset of physical stress, like thermal and traumatic injury [19] that modulates the immune response, being a good experimental model for understanding the relationship between physical exercise and pathogenesis of noninfectious systemic diseases [20]. In fact, it is able to modify immune cell distribution, phenotype [20], and cytokine production [6, 21]. Indeed, Terra and coworkers [22] demonstrated that aerobic exercise enhanced the production of IL-12 and TNF-*α* when stimulated by LPS or infected with *Leishmania major* (an intracellular protozoan), compared with the nonexercised control group [22]. Therefore, physical exercise is able to modulate the immune response against infectious and noninfectious stimuli.

Some studies suggest that physical exercise acts as an epigenetic inductor in peripheral leukocytes [23, 24]. Until now, the studies have focused primarily on the impact of acute exercise on methylation changes in PBMCs [24, 25]. Therefore, there is a relative lack of data on the effects of exercise on histone modifications in immune cells of obese individuals. Recently, our group has demonstrated that acute high-intensity interval exercise was able to increase the global HDAC activity in PBMC of obese men [26]. In this sense, there is no data on the influence of H4ac and HDAC2 status in PBMC of obese individuals in response to TLR4 agonist under basal and exercised conditions. Here, we evaluate the effect of acute strenuous exercise on epigenetic markers in LPS-stimulated PBMC, the cytokine production, and in the peripheral frequency of monocyte phenotypes of lean and obese individuals.

## 2. Methods

### 2.1. Subjects

Twenty physically active men took part in this study: ten lean (BMI 18.6 to 24.9 kg/m^2^) and ten obese level 1 (BMI 30.0 to 34.9 kg/m^2^). All participants were apparently healthy and generally physically active, but none engaged in physical training (based on an International Physical Activity Questionnaire-IPAQ with a score less than 1500 MET (minute/week)—https://www.ipaq.ki.se/downloads.htm). Participants were healthy, none smokers, age ranging from 20 to 40 years, and free of illness and injury, and they were not engaged in physical training programs for a period of six months prior to experimental trials. Exclusion criteria included autoimmune, cardiac, endocrine or metabolic diseases, acute and chronic infections, and under current drugs or dietary supplements with recognized impact on the immune system. After inclusion in the study, subjects completed a medical questionnaire and the Physical Activity Readiness Questionnaire. The Ethics Committee of the UFCSPA approved this study (protocol number 1.973.432), and all experimental procedures were performed according to the Declaration of Helsinki. All participants were informed about the study and signed the informed consent.

### 2.2. Preliminary Measurements

One week before the experimental trial, all participants went to the laboratory to estimate their body composition and cardiorespiratory fitness. During the trials, the temperature and humidity of the room were continuously controlled (18 to 24°C and humidity 50–70%). Prior to the analysis, participants were asked to fast for 12 h and to have a good night sleep of at least 8 h. The participants were strongly encouraged to avoid moderate to high energy expenditure activities 24 h before the cardiorespiratory test and the exercise trial. On this day, a fasting blood sample (4 mL) was collected into tubes without anticoagulant (BD, EUA), centrifuged (1000*g*), aliquoted, and frozen at −20°C to determine the metabolic profile (glucose and lipid profile).

A qualified professional performed all anthropometric evaluations, and all tests were performed three times and the average was used for analysis. Body mass (kg) and height (meters) were determined by a semi-analytical scale (Welmy, Santa Barbara D'Oeste, Brazil), with capacity for 200 kg and a stadiometer attached (Welmy, Santa Barbara D'Oeste, Brazil) with an accuracy of 0.1 kg and 0.005 cm, respectively. Waist (WC, cm), abdomen (AC, cm), and hip (HC, cm) circumferences were measured through an inelastic measuring tape. BMI (kg/m^2^) was defined as body mass (kg) divided by the square of height (m^2^).

An incremental exercise test was conducted to determine VO2 max using the breath-by-breath method and an open-circuit spirometry system (Quark CPET, COSMED, Rome, Italy). The progressive exercise test was performed on a treadmill and lasted for 8 to 12 min according to the recommendations of the American College of Sports Medicine (ACSM) [27]. The test ended if the participant reached volitional exhaustion, respiratory exchange ratio (RER) ≥ 1.15, or HR ≥ 95% of age-predicted maximum. VO2 max was determined when a plateau in the VO2 occurred when it is further increased by exercise intensity.

### 2.3. Experimental Design and Procedures

After the preliminary visit, participants arrived at the laboratory at 8:00 to perform a single session of strenuous exercise. One hour before the exercise, participants consumed a normal breakfast (average 300 kcal: orange juice, cake, banana, and yogurt). A 24 h diet recall interview was performed by a nutritionist to record the amount of food and beverage consumed 24 h before the exercise trial. Energy intake was determined using a dietary analysis software (DietWin, Porto Alegre, Brazil).

After that, they remained at rest during 5 minutes and a resting (rest) blood sample was taken from the antecubital vein using sterile vacutainer tubes (15 mL into K_3_EDTA tube) (Becton Dickinson (BD) Juiz de Fora, MG, Brazil). Then, they were required to perform a strenuous exercise consisting of stepping up and down from a step. The step height was individually adjusted to the height of each subject's femoral condyles. The stepping rhythm was paced acoustically at 60 beats per minute with same periods (1 s) for stepping up and down, respectively. When the participants were not able to maintain the required stepping rhythm, a 30 s recovery period was given to them. Following, they were required to recommence another set of the stepping cadence until fatigued and subsequently given another 30 s recovery period. This sequence was continued until complete exhaustion when even after a 30 s recovery the subject was not able to continue. Further venous blood samples (15 mL) were taken immediately after exercise. This protocol is based on the study of Saxton and coworkers [28]. For each subject, the total number of sets, stepping repetitions in each set, heart rate response, and perceived exertion, was recorded.

### 2.4. Peripheral Blood Mononuclear Cell (PBMC) Isolation and Stimulation with TLR4 Agonist

PBMC of lean and obese individuals was isolated from peripheral blood using Histopaque 1077 (Sigma, USA) as described by Bicalho et al. [29]. Plasma was collected and frozen at −20°C for biomarkers analysis as described below. PBMC viability was determined by trypan blue exclusion and the viability was always >95%. Then, the cells were washed and suspended in Roswell Park Memorial Institute-1640 medium (RPMI-1640, Sigma, USA) supplemented with 2 g/L sodium bicarbonate, 10% fetal bovine serum (FBS, Sigma-Aldrich, St. Louis, MO), 2% glutamine, and 100 U/mL penicillin–0.1 mg/mL streptomycin (Sigma-Aldrich, St. Louis, MO). Cells (1 × 10^5^/mL) were stimulated with a toll-like receptor 4 agonist (TLR4) at conditions previously established (LPS 10 ng/mL, 24 h, 5%CO_2_, 37°C) in 24-well plates in a final volume of 1 mL [30]. Then, the culture supernatants were collected, centrifuged (200*g*, 25°C, 10 min), and stored at −80°C for cytokine quantification, and PBMC were collected, washed with PBS 1x, and frozen at −80°C in a solution containing 90% FBS and 10% dimethyl sulfoxide (DMSO) for epigenetic analysis.

### 2.5. Cell Staining and Flow Cytometry

The frequency of classical noninflammatory (CD14^+^CD16^−^) and nonclassical proinflammatory (CD14^+^CD16^+^) circulating monocytes was determined in freshly isolated PBMCs of lean and obese individuals at rest and after strenuous exercise. Briefly, 2 × 10^5^ cells/mL was stained with monoclonal antibodies conjugated with specific fluorochromes: anti-human CD14-FITC (clone 61D3; BIOGEMS, USA) and anti-human CD16-PE (clone B73.1; BIOGEMS, USA). After 15 minutes of incubation, cells were resuspended in 0.4 mL of PBS-BSA 1% and analyzed by flow cytometry. Cell phenotype was acquired using CELLQuest Pro Software (BD Bioscience, USA) on a FACSCalibur flow cytometer (BD Bioscience, USA) equipped with a 15 mW argon laser-emitting light at a fixed wavelength of 488 nm. Fluorescent signals were collected in logarithmic mode (six-decade logarithmic amplifier). Monocytes were identified and gated according to their forward scatter (FSC) and side scatter (SSC) profiles. In monocyte gate, the percentage of CD14^+^CD16^−^ and CD14^+^CD16^+^ cells was determined based on fluorescence-1 (FL1-FITC) versus fluorescence 2 (FL2-PE) dot plots. Single color control tubes were used in each assay to account for spectral overlap. A minimal of 30,000 events of gated monocytes was acquired for analysis.

### 2.6. Leukocyte Counts and Levels of Metabolic Components

Total white cell count (WBC) were determined from vacutainer tubes containing EDTA (Becton Dickinson (BD) Juiz de Fora, MG, Brazil) in an automated analyzed ABX Micros60 (Horiba ABX, Kyoto, Japan). Fasting concentrations of glucose, total cholesterol, high-density lipoprotein (HDL), low-density lipoprotein (LDL), and triglycerides were determined in the serum by standard clinical laboratory methods, according to the recommendations of the Clinical and Laboratory Standards Institute (CLSI) guidelines in an automated biochemical machine (BS120, BioClin, Brazil). Circulating cortisol and leptin levels were determined by enzyme immunoassay method (Monobind Inc., USA) following the manufacturer's recommendations. Thiobarbituric acid-reactive substances (TBARS) are byproducts of lipid peroxidation and represent markers of oxidative stress. Concentrations of TBARS in plasma were determined spectrofluometrically following a previously described protocol [31] and expressed in MDA nmol/mL. Total thiol concentrations were measured in plasma using 5-5′-dithio-bis(2-nitrobenzoic acid) reagent [32].

Protein carbonyls were determined in plasma samples using the 2,4-dinitrophenolhydrazine (DNPH) spectrophotometric method described by Levine et al. [33]. Briefly, proteins of plasma samples (1 mL) were precipitated with 0.5 mL 10% trichloroacetic acid and centrifuged at 1500*g* for 5 min, and the supernatant was discarded. In the following, 0.5 mL of 10 mM 2.4-dinitrophenylhydrazine (DNPH) in 2 M HCl was added to precipitate protein and incubated at room temperature for 30 min. After incubation, 0.5 mL 10% TCA was added to the protein precipitate and centrifuged at 1800*g* for 5 min. The supernatant was discarded, the precipitate was washed twice with 1 mL ethanol/ethylacetate (1 : 1), and the supernatant was centrifuged out in order to remove the free DNPH. The precipitate was dissolved in 1.5 mL protein dissolving solution (2 g SDS and 50 mg EDTA in 100 mL 80 mM phosphate buffer, pH 8.0) and incubated at 37°C for 10 min. The color intensity of the supernatant was measured using a spectrophotometer at 370 nm. Carbonyl content was calculated using the molar extinction coefficient (21 × 10^3^ L·mol^−1^·cm^−1^), and the results are expressed as nmol/mg protein.

### 2.7. Global Histone H4 Acetylation (H4ac) and Histone Deacetylase 2 (HDAC2) Activity in PBMC

Histone extracts from PBMCs were prepared using a total histone extraction kit (EpiGentek) according to the manufacturer's protocol. H4ac levels in PBMCs were determined using the Global Histone H4 Acetylation Assay Kit (Colorimetric Detection, catalog number P-4009, EpiQuik, USA) according to the manufacturer's instructions and expressed as ng/mg protein. The protein concentration of each sample was measured by the Coomassie Blue method using bovine serum albumin as standard. The protein content of HDAC2 was measured using colorimetric EpiQuick HDAC2 assay kits (EpiGentek) according to the manufacturer's protocol. Results were calculated using a standard curve and expressed as ng/mg of protein.

### 2.8. Cytokines and Leptin Quantification

The concentrations of TNF-*α* (eBioscience, USA), IL-6 (eBioscience, USA), and IL-8 (PeproTech, USA) into the supernatants of LPS-stimulated or nonstimulated cells were quantified by enzyme-linked immunosorbent assay (ELISA). Serum levels of leptin were also evaluated using a commercial kit (PeproTech, USA). The intra-assay coefficient variation was always <7.5%.

### 2.9. Statistical Analysis

The SPSS 20.0 (IBM Inc., USA) software was used for statistical analysis. Data are reported as the mean ± Standard deviation (SD). The Shapiro-Wilk test was used to evaluate the normality of the data. Demographic and anthropometric characteristics were compared between groups through an independent Student's *t*-test. A two-way analysis of variance was used with group (obese versus lean) and time (rest and after exercise) as factor with Bonferroni's correction to multiple analysis. Correlations were performed by Pearson's correlation coefficient. *p* values <0.05 were considered statistically significant.

## 3. Results

### 3.1. Demographic and Metabolic Characteristics of Subjects and Nutritional Habits

Participant's characteristics are outlined in Table 1. As expected, obese individuals had higher body mass (*p* < 0.001), body mass index (*p* < 0.001), abdominal circumference (*p* < 0.001), waist circumference (*p* < 0.001), hip circumference (*p* < 0.001), and waist : hip ratio (*p* < 0.001) than lean individuals. The groups were homogeneous with respect to age, height, and cardiorespiratory fitness (*p* > 0.05), and no difference was observed regarding the serum glucose, total cholesterol, HDL, LDL, and triglycerides levels (*p* > 0.05).

As demonstrated in Table 2, obese individuals had higher total energy intake (*p* = 0.02) and amount of lipids (%, *p* = 0.025; g, *p* = 0.029) consumed than lean individuals. Moreover, differences were found in the amount of protein consumed between lean and obese individuals (%, *p* = 0.036; g, *p* = 0.041).

### 3.2. Exercise Intensity Markers

The duration of exercise was approximately 1275 ± 294 s in the lean group and 1023 ± 342 s in the obese group (*p* = 0.029). The average heart rate was 165.78 ± 6.72 bpm (88.3 ± 30% of HRMax) in the lean and 163.29 ± 3.97 (92.6 ± 28% of HRMax) in the obese group (*p* > 0.05). The average exertion perceived in the lean was 17.66 ± 0.89 and 18.72 ± 0.26 in the obese group (*p* > 0.05). Obese individuals performed significantly lower total number of repetitions than lean (482 ± 62.2 versus 572.2 ± 34.7; *p* = 0.035).

### 3.3. Systemic Biomarkers in Response to Strenuous Exercise in Lean and Obese Subjects

At rest, in obese individuals, the cortisol (*p* = 0.02) and leptin (*p* < 0.001) levels were significantly higher compared with those found in lean individuals. In the lean group, the strenuous exercise induced a significant increase in WBC count (*p* = 0.01), cortisol (*p* = 0.002), protein carbonyls (*p* = 0.042), and TBARS (*p* = 0.034) levels and decreased the content of total thiols in plasma (*p* = 0.03). Similarly, obese individuals presented an elevation in WBC count (*p* = 0.004), cortisol concentrations (*p* = 0.001), protein carbonyls (*p* = 0.026), and TBARS response (*p* = 0.025) and a reduction in total thiol concentrations (*p* = 0.032) after strenuous exercise. In addition, after the exercise, plasmatic levels of cortisol (*p* = 0.02), leptin (*p* < 0.001), and protein carbonyls (*p* = 0.042) were higher in the obese group compared with the lean group (Table 3).

### 3.4. Effect of Strenuous Exercise in Classical and Nonclassical Monocytes Phenotype in Lean and Obese Individuals

The percentage of noninflammatory CD14^+^CD16^−^ and proinflammatory CD14^+^CD16^+^ monocytes freshly isolated is presented in Table 4. At rest, the percentage of proinflammatory monocytes tended to be higher in obese compared with lean individuals (*p* = 0.06), but no differences were observed in noninflammatory monocytes between groups (*p* > 0.05). However, the strenuous exercise induced a significant increase in nonclassical proinflammatory monocytes in obese (*p* = 0.016) as well as in lean subjects (*p* < 0.001). At the end of the exercise, the percentage of CD16^+^ monocytes remained higher in obese compared with lean individuals (*p* = 0.022). No significant difference was observed in CD16^−^ monocytes in both groups (*p* < 0.05).

### 3.5. Effects of Exercise Stress and Immunological Challenge on Levels of Histone H4 Acetylation and HDAC2 Activity in PBMC from Obese Individuals

**Figure 1:**
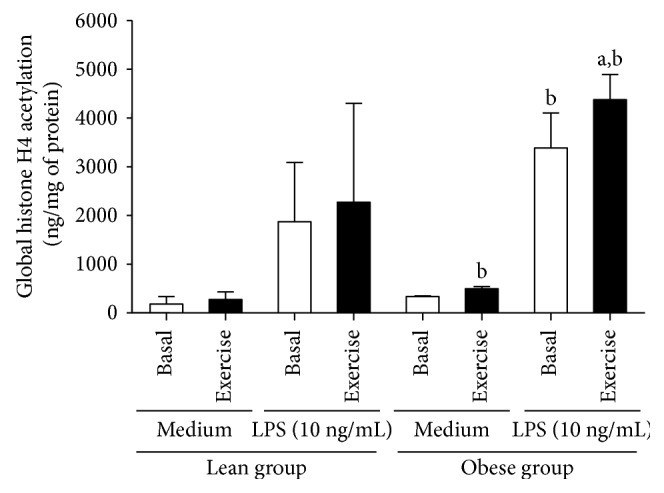
Global histone H4 acetylation levels in nonstimulated and LPS-stimulated PBMC isolated from lean and obese individuals in basal and exercised conditions. Data presented as mean ± SD. ^a^Statistical difference from basal values (*p* < 0.05). ^b^Statistical difference compared to lean group (*p* < 0.05).

Figure 1 presents the effects of strenuous exercise on the levels of global H4ac in PBMC of lean and obese subjects before and after LPS challenge. PBMCs isolated from nonexercised lean and obese individuals presented similar levels of global H4ac when incubated *in vitro* in the presence of medium (lean 182.17 ± 152.55 ng/mg of protein; obese 336.33 ± 15.84 ng/mg of protein; *p* > 0.05). However, the stimulation of PBMC with LPS caused a significant hyperacetylation of H4ac in cells of obese (3382.89 ± 718.21 ng/mg of protein) compared with cells from of individuals (1873.07 ± 1216.92 ng/mg of protein) (*p* = 0.015). Moreover, in obese individuals, the acute strenuous exercise increased the global H4ac in both nonstimulated (*p* = 0.01) and LPS-stimulated (*p* = 0.04) PBMC. In contrast, in the lean group, the exercise was not able to modulate the levels of H4ac in PBMC even after the challenge with LPS (*p* > 0.05).

**Figure 2:**
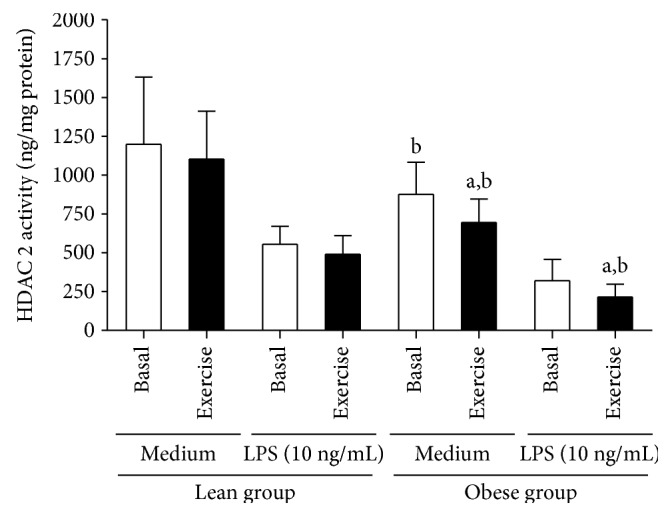
Global HDAC2 activity in nonstimulated and LPS-stimulated PBMC obtained from lean and obese individuals in basal and exercised conditions. Data presented as mean ± SD. ^a^Statistical difference from basal values (*p* < 0.05). ^b^Statistical difference compared to the lean group (*p* < 0.05).

Regarding the histone deacetylases, we evaluated the effect of strenuous exercise on HDAC2 activity in PBMC of obese individuals under basal and LPS-stimulated conditions. At baseline, lean individuals had higher activity of HDAC2 in LPS-stimulated PBMC (554.42 ± 116.63 ng/mg of protein) than obese individuals (320.14 ± 136.71 ng/mg of protein) (*p* = 0.005). At baseline, LPS-stimulated PBMC of lean presented higher HDAC2 activity (554.42 ± 116.63 ng/mg of protein) compared with those cells of obese individuals (320.14 ± 136.71 ng/mg of protein) (*p* = 0.005). No effect of exercise was observed on HDAC2 activity in both nonstimulated and LPS-stimulated PBMC of the lean group (*p* > 0.05). On the other hand, a significant reduction on HDAC2 activity in PBMC was observed after exercise in both nonstimulated (*p* = 0.022) and LPS-stimulated (*p* = 0.030) conditions in obese groups (Figure 2).

### 3.6. Cytokine Production

**Figure 3:**
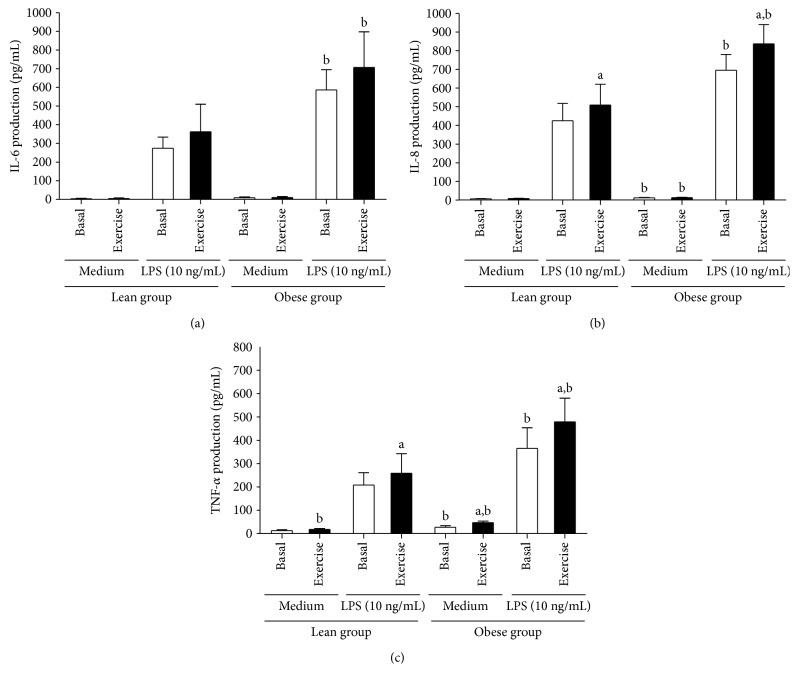
Production of IL-6 (a), IL-8 (b), and TNF-*α* (c) by nonstimulated and LPS-stimulated PBMC obtained from lean and obese individuals in basal and exercised conditions. Data presented as mean ± SD. ^a^Statistical difference from basal values (*p* < 0.05). ^b^Statistical difference compared to the lean group (*p* < 0.05).

The production of IL-6 (Figure 3(a)), IL-8 (Figure 3(b)) and TNF-*α* (Figure 3(c)) by PBMC isolated from lean and obese subjects, at rest and exercised conditions, was evaluated after *in vitro* stimulation with LPS. At rest, compared with lean, PBMCs of obese individuals displayed an inflammatory profile in both nonstimulated and LPS-stimulated conditions. In this line, the production of IL-8 (*p* = 0.024) and TNF-*α* (*p* = 0.015) by nonstimulated cultured PBMC, as well as the production of IL-6 (*p* < 0.001), IL-8 (*p* < 0.001), and TNF-*α* (*p* < 0.001) by LPS-stimulated cells was higher in obese compared with the lean group. In the lean group, PBMCs isolated from exercised individuals when cultured in the presence of medium (control), compared with nonexercised condition, produced a high amount of TNF-*α* (*p* = 0.024). In addition, when cells of exercised lean were stimulated *in vitro* with LPS, compared with the basal condition, a significant increase in IL-8 (*p* = 0.039) and TNF-*α* (*p* = 0.039) was observed. In the obese group, a significant increase in TNF-*α* (*p* = 0.01) was observed in nonstimulated cells after exercise compared to the rest condition. Moreover, a significant increase in LPS-stimulated condition, relative to IL-8 (*p* = 0.02) and TNF-*α* (*p* < 0.001) production, after exercise in obese individuals was observed. In addition, at the end of the exercise, significant differences between groups (lean and obese) were found in the levels of IL-8 (*p* = 0.015) and TNF-*α* (*p* < 0.001) secreted by nonstimulated PBMC and IL-6 (*p* = 0.003), IL-8 (*p* = 0.001), and TNF-*α* (*p* < 0.001) released by LPS-stimulated cells.

### 3.7. Correlations

Values of basal global H4ac in LPS-stimulated PBMC have a positive correlation with body mass (*r* = 0.63; *p* = 0.01), BMI (*r* = 0.63; *p* = 0.01), AC (*r* = 0.66; *p* = 0.01) and WC (*r* = 0.57; *p* = 0.03), and nonclassical proinflammatory monocytes (*r* = 0.53; *p* = 0.05). In addition, postexercise, the global H4ac was positively correlated with body mass (*r* = 0.57; *p* = 0.03), BMI (*r* = 0.54; *p* = 0.04), AC (*r* = 0.55; *p* = 0.04), and WC (*r* = 0.56; *p* = 0.03). Moreover, when we calculated the delta value (postexercise−basal values) of epigenetic and cytokine markers (TNF-*α*) to verify possible correlations between these variables induced by acute exercise, a positive correlation was found between delta value of global H4ac and delta value found for TNF-*α* production induced by LPS stimulation (*r* = 0.75; *p* = 0.002) (Table 5).

Regarding basal HDAC2 activity on LPS-stimulated PBMC, these parameters had negative correlations with body mass (*r* = −0.60; *p* = 0.02), BMI (*r* = −0.70; *p* < 0.001), AC (*r* = −0.69; *p* < 0.01), WC (*r* = −0.79; *p* < 0.01) and nonclassical proinflammatory monocytes (*r* = −0.63; *p* = 0.01). The HDAC2 activity in LPS-stimulated PBMC obtained from exercised individuals also presented negative correlations with body mass (*r* = −0.70; *p* < 0.01), BMI (*r* = −0.84; *p* < 0.001), AC (*r* = −0.69; *p* < 0.01), and nonclassical proinflammatory monocytes (*r* = −0.66; *p* < 0.001) (Table 6).

## 4. Discussion

To the best of our knowledge, this is the first study describing the influence of strenuous exercise on the acetylation status of histone H4 and HDAC2 activity in PBMC of obese individuals. Our results showed that, at rest, LPS-stimulated PBMC of obese individuals presented high H4ac levels and low HDAC2 activity compared with lean. In obese men, the acute strenuous exercise increased the global H4ac and reduced the HDAC2 activity in both nonstimulated and LPS-stimulated PBMC. Moreover, the exercise induced a significant increase in nonclassical proinflammatory monocytes and a marked increase in the production of proinflammatory cytokines, IL-8 and TNF-*α*, especially in obese men. Taken together, these results suggest that the excess of fat mass alters the acetylation events and the chromatin remodeling in PBMCs.

Obese individuals reported higher total energy intake and lipid consumption when compared to lean subjects. High-fat diets are reported to induce changes in proinflammatory gene expression and key genes related to lipid metabolism in PBMC [34]. The acute and long-term lipid consumption correlates with the upregulation of genes related to inflammation such as CD16A, MCP-1, IL-6, TNF-*α*, and p65 in PBMC and adipose tissue [35]. In fact, obese mice treated with hypercaloric and high-fat diet had altered cytokine expression in peritoneal macrophages after LPS stimulation [36]. Thus, the higher amount of lipid consumption and total energy intake by obese individuals impacts on proinflammatory status of PBMC. However, we highlight that in our study, the obese group did not present systemic lipid metabolism deregulation, as demonstrated by the absence of changes in lipid parameters compared to lean.

The epigenetic modulation across altered DNA methylation and histone modification have been linked to the dysfunction of the immune system in obese individuals [7]. In our study, the chronic low-grade inflammation observed in obesity was associated with hyperacetylation of histone H4 in PBMC. Previous studies have also shown that leukocytes from obese individuals presented a proinflammatory status [4, 5, 37]. In this sense, Ghanim and coworkers [4] described that PBMC from obese individuals had deregulated NF-*κ*B expression and proinflammatory cytokines expression than matched lean controls. In addition, monocytes of obese individuals present an M1 phenotype, characterized by the CD14^+^CD16^+^ expression, elevated expression of TLR-4 and TLR-8, and a large amount of TNF-*α* and IL-6 production, after PBMC stimulation with nonesterified fatty acids [38, 39]. Collectively, these data are in accordance with our results regarding the proinflammatory phenotype of PBMC of obese men, which produced high amounts of proinflammatory IL-6, IL-8, and TNF-*α*. In this way, we found positive correlations between basal H4ac levels in LPS-stimulated PBMCs and anthropometric markers (i.e., BMI, AC, and WC) that confirm the close relationship between obesity and histone H4 hyperacetylation.

Interesting, both M1 phenotype and enhanced NF-*κ*B expression are dependent on H4ac levels [12, 13]. In this sense, previous studies demonstrated that the proinflammatory polarization of human monocytes by interferon gamma or LPS plus TNF-*α* is regulated by H4ac status [13, 40]. Moreover, Tsaprouni et al. [15] described that the enhancement of gene transcription of IL-8 and TNF-*α* is widely dependent on histone 3 and 4 acetylation, which enhances the inflammatory gene transcription after *in vitro* LPS stimulation. Indeed, the immunosuppressive action of glucocorticoids involves deacetylation of histone H4 in proinflammatory promoter genes via augmentation of HDAC2 activity [19].

However, our study revealed that the higher levels of cortisol found in the obese group have no effect in acetylation-deacetylation events in PBMC of obese individuals. In fact, the immunosuppressive action of cortisol in LPS-stimulated monocytic production of TNF-*α* was inhibited in obese subjects [41]. Thus, the impaired cortisol action on PBMC of obese individuals may contribute to the enhancement in proinflammatory cytokine production and histone H4 hyperacetylation in obesity. On the other hand, *in vitro* data revealed that leptin increased the activation of MAPKs/NF-*κ*B pathway through acetylation of histone H4 [42]. As the expansion of adipose tissue in obesity is accompanied by the augmentation of leptin, we hypothesized that leptin may play a key role in H4ac status in PBMC of the obese.

In the present study, *in vitro* stimulation with LPS significantly increased the H4ac levels and decreased HDAC2 activity in PBMCs from both lean and obese individuals. In fact, H4ac is widely involved in the gene transcription after LPS stimulation [43]. Mechanistically, the decreased HDAC2 activity may occur through oxidative stress [44] or polarizing signals like cytokines or endocrine factors [12, 43]. In fact, HDAC2 depletion can lead to the increase of inflammatory gene expression via free NF-*κ*B [45]. Thus, the decreased HDAC2 activity in obesity may be a potential target for pharmacological or exercise-based therapies.

In this study, obese individuals presented low tolerance to exercise, demonstrated through short duration of exercise bout and low number of repetitions before reaching a state of exhaustion compared to lean individuals. However, similar HR response and perceived exertion were found at the end of the exercise between groups. In fact, excess body fat increases the oxygen cost and cardiorespiratory load during submaximal or exhaustive exercises [46]. On the other hand, while the cardiac output and stroke volume in response to exhaustive exercise seems to be altered in obese individuals, the oxygen consumption and HR response were not altered indicating that the ability of the cardiorespiratory system to transport oxygen to the exercised muscle during strenuous exercise was not impaired in obese individuals [47]. Thus, while obesity may have an impact on exercise tolerance, the excess of body fat has little impact on cardiovascular response as indicated by similar HR response and the percentage of HR maximal reached immediately after strenuous exercise.

Acute strenuous exercise also increased the oxidative damage markers (TBARS and protein carbonyls) and caused a reduction in total thiols content in both groups. Increases in ROS production during and after exercise can occur through different mechanisms, including increased oxidative phosphorylation and electron leakage in the electron transport system, activation of xanthine oxidase pathway, NADPH oxidase activity in peripheral phagocytes, as well as, impaired calcium homeostasis, mechanical stress, and ischemia-reperfusion events [48]. Moreover, TBARS and protein carbonyl levels seem to be responsive to maximal intensity exercise and the degree of muscle damage induced by exercise, but the same result was not observed after a submaximal intensity exercise [49]. After exercise, obese individuals displayed higher concentrations of protein carbonyls compared to lean. Proteins are major targets for ROS, and an increased protein oxidation has been previously reported [49]. Obesity is associated with higher levels of oxidative stress that causes oxidative damage to proteins, lipids, and DNA molecule. In addition, sedentary obese individuals are more susceptible to oxidative damage due to reduced enzymatic antioxidant activity [48]. In this way, future studies should be conducted in order to evaluate the acute effects of strenuous exercise on antioxidant enzymes (i.e., catalase, superoxide dismutase) of obese individuals.

This study sheds light on the exercise-induced epigenetic changes on immune cells. We demonstrated that strenuous exercise induced an increase in global H4ac and decrease in HDAC2 activity in LPS-stimulated PBMC of obese individuals. In our study, the absence of changes in HDAC2 activity and global H4ac status in both nonstimulated and LPS-stimulated PBMC of lean individuals after strenuous exercise corroborates previous reports on epigenetic changes in PBMC from healthy individuals in response to acute exercise [50, 51]. On the other hand, the densiometric units from acetylated histone 4 on lysine 5 (H4K5) were significantly elevated after a half marathon in both cancer patients and healthy controls [52]. Interestingly, a single bout of vigorous exercise enhanced NF-*κ*B DNA-binding activity in PBMC from healthy individuals [53]. In addition, recently, we demonstrated that a single bout of high-intensity interval exercise (HIIE) was able to elevate the global activity of HDAC in PBMC of obese individuals [26] and the systemic levels of IL-10 and TGF-*β*, suggesting that HDAC plays an important role in the immunoregulatory activity of HIIE. Exercise-induced epigenetic changes in PBMC seems to be exercise-dependent, since acetylation-deacetylation events or DNA methylation returned to basal values 24 h after HIIE or moderate-continuous exercise [26, 50], while strenuous exercise was able to maintain higher histone acetylation status 24 h after bout [52]. However, most studies did not evaluate the epigenetic changes 24 h after exercise [25, 51]. Thus, we believed that the histone modifications in PBMC and the inflammatory response are dependent of the type and intensity of exercise.

The BMI status is another important key regarding histone modifications in response to exercise. In our study, a positive correlation was found between postexercise global H4ac levels and anthropometric markers such as body mass, BMI, and trunk circumferences related to central adiposity. In this line, Lavratti et al. [23] demonstrated that a reduction in global H4ac of phytohemagglutinin-stimulated PBMC of schizophrenic patients was accompanied by weight loss following 90 days of combined strength plus endurance exercise training. Thus, the reduction in histone acetylation status altered the production of IL-6, IFN-*γ*, and TNF-*α* after moderate exercise training [8, 23].

As expected, we found that strenuous exercise increased the proinflammatory IL-8 and TNF-*α* production by PBMC of obese and lean individuals after *in vitro* treatment with LPS. Moreover, in the obese group, the peripheral frequency of nonclassical proinflammatory monocytes was higher after exercise compared to the lean group. Interestingly, this proinflammatory status enhanced by strenuous exercise in obese men was accompanied by increase acetylation status of histone H4 in PBMC. Reinforcing this, a strong positive correlation between delta values of TNF-*α* and H4ac levels in PBMC of obese individuals was found. Elevations in the production of LPS-induced proinflammatory cytokines after exhaustive exercise were previously reported [21, 54, 55]. Moreover, strenuous exercise induces a significant decrease in corticosteroid sensitivity in peripheral immune cells [56]. This may indicate that impaired cortisol immunosuppression action after exercise can contribute to the increase of H4ac in LPS-stimulated PBMC and the higher cytokine production after the bout. In addition, the elevation in protein carbonyls after exercise in obese individuals may contribute to H4ac and proinflammatory response. Indeed, previously *in vitro* data revealed that the treatment with hydrogen peroxide induces IL-8 promoter hyperacetylation and reduces the HDAC activity in bronchial epithelial cells [57]. Thus, H4ac status alters the cell phenotype and cytokine production pattern of both innate and adaptive immunity [16, 49].

Some studies have demonstrated that an acute bout of strenuous exercise induced a significant increase in nonclassical monocyte proportion in both healthy [58–60] and obese individuals [61, 62]. It was postulated that the greater catecholaminergic response, the degree of muscle damage and oxidative stress response mediates the proinflammatory monocyte mobilization. In fact, CD14^+^CD16^+^ monocytes showed a rapid and transient increase after epinephrine infusion in healthy men [63]. On the other hand, some reports described that CD16^+^ monocytes are preferentially removed from the bloodstream in healthy individuals following 1 h of recovery after exercise [60]. Thus, fat mass may impact on selective CD14^+^CD16^+^ monocyte tissue mobilization after strenuous exercise. Contributing to this, leptin promotes the development and selective mobilization of CD16^+^ monocytes and the *in vitro* TNF-*α* production by stimulated monocytes [64]. Thus, the histone H4 hyperacetylation status after strenuous exercise contributes to a polarization to the nonclassical monocyte phenotype in obese individuals.

## 5. Conclusion

In conclusion, this study provides evidence that a single session of strenuous exercise induces an imbalance between H4ac and HDAC2 activity and increases the proinflammatory response. Collectively, the data suggests an influence of epigenetic machinery in the immunomodulatory effects promoted by exercise.

## Figures and Tables

**Table 1:** Demographic and metabolic characteristics of participants.

	Lean (*n* = 10)	Obese (*n* = 10)
Age (years)	47.71 ± 6.96	48.28 ± 4.19
Height (meters)	1.72 ± 0.07	1.74 ± 0.08
Body mass (kg)	66.67 ± 9.18	101.47 ± 16.25^∗^
BMI (kg/m^2^)	22.28 ± 1.91	32.98 ± 2.49^∗^
WC (cm)	70.71 ± 5.02	99.57 ± 8.94^∗^
AC (cm)	79.20 ± 3.37	99.57 ± 8.94^∗^
HC (cm)	83.62 ± 3.21	103.46 ± 13.21^∗^
WHR	0.83 ± 0.04	0.96 ± 0.02^∗^
VO_2Peak_ (mL.kg^−1^.min^−1^)	42.15 ± 5.49	38.47 ± 7.16
Glucose (mg/dL)	75.8 ± 6.70	82.7 ± 3.09
Total cholesterol (mg/dL)	168.7 ± 27.90	193.57 ± 40.10
HDL (mg/dL)	58.23 ± 10.25	52.17 ± 14.84
LDL (mg/dL)	116.47 ± 19.74	134.12 ± 34.27
Triglycerides (mg/dL)	80.76 ± 17.64	109.76 ± 41.15

Data presented as mean ± SD. AC: abdominal circumference; BMI: body mass index; HDL: high-density lipoprotein; LDL: low-density lipoprotein; WC: waist circumference; HC: hip circumference; WHR: waist : hip ratio. ^∗^Statistical difference between lean and obese groups (*p* < 0.05).

**Table 2:** Reported macronutrient intake in 24 h diet recall between groups.

	Lean	Obese
Total energy intake (kcal)	1972.36 ± 552.99	2524.05 ± 886.01^∗^
Carbohydrates (%)	50.84 ± 9.71	52.85 ± 6.63
Carbohydrates (g)	281.95 ± 61.22	285.37 ± 91.75
Protein (%)	24.43 ± 6.87	18.12 ± 4.19^∗^
Protein (g)	162.8 ± 41.90	145.4 ± 38.70^∗^
Lipids (%)	26.73 ± 5.56	30.73 ± 8.91^∗^
Lipids (g)	81.1 ± 30.45	90.36 ± 26.72^∗^

Data presented as mean ± SD. ^∗^Statistical difference between lean and obese groups (*p* < 0.05).

**Table 3:** Systemic biomarkers in lean and obese individuals at rest and after strenuous exercise.

	Time point	Lean	Obese
WBC (10^3^/mm^3^)	Rest	6.45 ± 1.50	6.58 ± 2.59
	Exercise	8.32 ± 2.42^a^	8.61 ± 3.21^a^
Cortisol (*μ*g/dL)	Rest	19.61 ± 4.05	31.02 ± 4.07^b^
	Exercise	22.91 ± 4.51^a^	36.66 ± 2.38^a,b^
Leptin (pg/mL)	Rest	22.15 ± 7.90	57.58 ± 14.33^b^
	Exercise	22.97 ± 8.45	57.08 ± 11.26^b^
TBARS (nmol/mL)	Rest	1.65 ± 0.52	1.72 ± 0.37
	Exercise	2.45 ± 0.59^a^	2.67 ± 0.48^a^
Protein carbonyl (nmol/mg protein)	Rest	1.25 ± 0.39	1.44 ± 0.43
	Exercise	1.67 ± 0.27^a^	1.98 ± 0.36^a,b^
Total thiol (*μ*mol/mL)	Rest	1.13 ± 0.21	1.18 ± 0.17
	Exercise	0.83 ± 0.19^a^	0.78 ± 0.26^a^

Data presented as mean ± SD. ^a^Statistical difference compared to rest values (*p* < 0.05). ^b^Statistical difference between groups (*p* < 0.05).

**Table 4:** Peripheral frequency of monocyte subsets in lean and obese individuals at rest and exercised conditions.

	% CD14^+^CD16^−^ (noninflammatory)		% CD14^+^CD16^+^ (proinflammatory)	
	Rest	Exercised	Rest	Exercised
Lean	82.31 ± 5.60	80.97 ± 8.31	11.16 ± 2.36	12.76 ± 2.21^a^
Obese	80.81 ± 2.76	77.32 ± 9.01	15.55 ± 5.14^b^	19.92 ± 5.91^a,b^

Data presented as mean ± SD. Values are expressed as the percentage of all gated CD14+ monocytes. ^a^Statistical difference compared to rest values (*p* < 0.05). ^b^Statistical difference between groups (*p* < 0.05).

**Table 5:** Correlation of H4ac status of LPS-stimulated PBMC and anthropometric, proinflammatory monocyte, and delta (Δ) value of TNF-*α* before and after exercise.

	Levels of H4ac in LPS-stimulated PBMC (ng/mg of protein)		
	Before	After	Δ (after-before)
Body mass (kg)	0.63 (0.01)	0.57 (0.03)	N.S.
BMI (kg/m^2^)	0.63 (0.01)	0.54 (0.04)	N.S.
AC (cm)	0.66 (0.01)	0.55 (0.04)	N.S.
WC (cm)	0.57 (0.03)	0.56 (0.03)	N.S.
CD14^+^CD16^+^ (%)	0.53 (0.05)	N.S.	N.S.
ΔTNF-*α*	N.S.	N.S.	0.75 (*p* = 0.002)

Pearson's Correlation presented as *r* (*p* value). *p* values were considered significant when ≤0.05. AC: abdominal circumference; BMI: body mass index; TNF-*α*: tumour necrosis factor-alpha; WC: waist circumference; N.S.: nonsignificant correlation.

**Table 6:** Correlation of HDAC2 activity of LPS-stimulated PBMC and anthropometric and monocyte subset before and after exercise.

	HDAC2 activity in LPS-stimulated PBMC (ng/mg of protein)	
	Before	After
Body mass (kg)	−0.60 (0.02)	−0.70 (<0.01)
BMI (kg/m^2^)	−0.70 (<0.001)	−0.84 (<0.001)
AC (cm)	−0.69 (<0.01)	−0.69 (<0.01)
WC (cm)	−0.79 (<0.01)	N.S.
CD14^+^CD16^+^ (%)	−0.63 (0.01)	−0.66 (<0.001)

Pearson's correlation presented as *r* (*p* value). *p* values were considered significant when ≤0.05. AC: abdominal circumference; BMI: body mass index; WC: waist circumference; N.S.: nonsignificant correlation.
